# Annual Address

**Published:** 1897-02

**Authors:** James M’Manus

**Affiliations:** Hartford, Conn.


### ANNUAL ADDRESS.[1]

BY JAMES M'MANUS, D.D.S., HARTFORD, CONN.

At the annual meeting of the Connecticut Valley and the New England Dental Societies, held in Worcester, Mass., October 23, 24, and 25, 1895, each society, after mature deliberation, voted to disband and the members of both societies to again unite to form a new society. The pioneer missionary work done by these societies is in part on record in the dental journals, while memory alone will recall the many dear old friends and pleasant gatherings for thirty-three years past that will linger long in the hearts of the few that are left of the original members of the disbanded societies.

With best wishes for the continued success of the new organization, the money left in the hands of the treasurers of the old societies was passed over as a christening present to the young Northeastern Dental Association. We have said good-by to the old societies, and to-day the new one is given a hearty welcome,

---

[1] Delivered before the Northeastern Dental Association, held at Springfield, Mass., October 21, 1896.

realizing, as we all must, that the new departure means, if anything, a broader and fuller recognition of the demands of the dental profession on each and every member in practice to-day in the New England States.

It has been said by several well-meaning men that dental societies are not now needed in these progressive days, and that under the dental laws, now in force in every State in the Union, the State society alone was the one to be built up and fostered. If dental laws are to be properly enforced, and they should be, the dental examiners should and must have the backing and moral support of the best men in the profession in each State. I have said the best men, for every practitioner has it in his power to be called one of the best men in his profession if he so wills it, and every young man especially should aim to gain such a record with his professional associates; but all young men and old should be enrolled as members of their State society and take an active interest in society work. They should strive to promote the welfare of their society, and their best judgment and influence should be exerted with the appointing power in their State to have only such men selected as dental examiners as are qualified both in head and heart to fill the responsible position acceptably to the profession and for the best interests of the public. The laws of the State and the society imply that care should be exercised in taking students, as it finally rests with the State examiners to grant or refuse a license to practise in many of the States; therefore only those that give promise of gifts and qualifications that would make competent dentists should be recommended to the different colleges for instruction and graduation.

Our plain duty, then, is first to build up our State societies, practically, theoretically, scientifically, and, what is of great, very great importance, socially, we need to know each other better than we do, for the social side of society gatherings brings out the best that is in us professionally. I have heard the remark made by some few men at society meetings, "that they did not learn anything at them." A very unfortunate admission for them to make, for if they did not hear or see anything of interest and value, it must have been because they were neither giving attention to the clinics nor listening to the papers that were read and the discussions that followed. How any man interested in a special line of work can fail to get new ideas and valuable suggestions while listening to a discussion by fellow-workers it is hard for me to conceive. If, before attending a dental meeting, we would recall the old maxim "that it is better to give than to receive," and each one of us go prepared and willing to take a part in the discussions and talks on office practice, our meetings might be made even much more interesting and instructive than they have been in the past. The men that built up the old societies of the country were full of that spirit. They gave their time, money, and earnest work freely to help each other. They gave their experience and told of their failures. They demonstrated their way of operating, and exhibited their pet instruments and asked for the introduction of new and better ones. They spurred each other on to study and write papers for the society and for the journals, in this way interesting those outside of society membership. Those that were present at the meetings when Drs. Taft and Atkinson visited the New England societies, in 1863, by request of the American Dental Association, will remember the interest they awakened and the new life given to society work by their advice and counsel. To the good work done by the societies of this country in the past is due the high position that American dentistry has reached to-day in the estimation of the people of the civilized world. "Men may come and men may go," but ideal competent dentists are needed now and will be for all time. Education, study, and practice will not completely accomplish everything. There is always a need for broader knowledge, and that can best and most pleasantly be acquired by attending the post-graduate schools held by the State and national societies each year. We are now restricted as practitioners by State laws, but I do not think the intention was to build a Chinese wall, cutting us off from social and professional intercourse with our friends in other States. The frequent union meetings held in the past tell plainly that we all desire to see and hear men from other States, and all are benefited by attending these meetings.

The Southern and American Dental Associations have had under consideration for some time past the advisability of uniting to form one national delegated society. Other plans have been suggested for a new order of district organizations throughout the country. What may result from these different movements remains to be seen. The disbandment of the old societies last year and the formation of the Northeastern Dental Association, to include all New England dentists, marks the first forward movement for a higher development of society work in this country. To carry on the work successfully will require the co-operation of every society in this district. Mutual arrangements should be made to hold the annual society meetings in the months of April, May, and June, so that all those who wish to visit the different State meetings can do so. Each State society should strive for a larger membership, and a more active interest must be taken by the individual members to make the meetings interesting and profitable, so that they will be anxiously and pleasantly looked forward to. Dental meetings properly conducted are in reality special schools for instruction, where one may get valuable advice as to the treatment of special cases, and technical instruction, both orally and through clinics, as to the various methods of operating under questionable conditions. Time should be set apart for relating incidents of office practice and for the practical talks that would follow, so that all may profit by hearing each other's experience. The young men should be encouraged to write papers and to take part in the discussions, for teachers and writers will be fully as much needed in the future as they have been in the past. That society meetings are the best training-schools to develop and bring to the front men that are best qualified to teach and write will be admitted by all who have watched the career of the successful writers and teachers of to-day.

There has been a great deal written and said about the great advance made by dentists and dentistry in the past fifty years. It is well to reflect and remember that all that has been gained must be credited to the self-sacrificing efforts of a very few earnest men, whose names you will all readily recall. We boast of the record of the past fifty years, forgetting the fact that it would be very difficult to give the names of fifty men (one for each year) worthy to be put on the roll of honor for advanced professional work, either as writers or teachers. When you recall the criticism that has been made so frequently of late years on the business management of the colleges, it would be well to remember what an immense professional debt we all owe to the men who have been connected with the colleges in the past and at the present time. Many of them have given years of earnest work as teachers, getting little, often no pay for their time. They have been on call in season and out of season to give lectures and talks at society meetings, without even being offered any compensation. A few among them have written exhaustive works on dentistry and others valuable text-books, while the current literature of dentistry has been mainly written by a few men who were more or less intimately connected with the colleges. These men should be honored now for the great work they have accomplished; they deserve and should be given the warmest thanks and support of the profession. The captious and often unjust criticism made by a few over the alleged mismanagement of some colleges should be listened to as we would to a wind squall,—soon over and sooner forgotten; for if there have been faults of management in any of them, they will in due time be remedied. If the individual members of the profession will see to it that only good, earnest students are recommended to the colleges, there will be little or no trouble in the future regarding the management of them. Owing to the laws now in force in the different States, the public are led to believe that all the dentists now in practice are properly qualified; for the laws are stringent and the dental examiners are supposed to be capable and thorough in their examinations. All dentists before the law stand equal, and for this reason we believe that the future of dentistry depends on society membership and society work. What has been done for dentistry in the past (aside from mechanical inventions and appliances) by men outside of society membership? and what better test can be had in the future of a man's qualifications than his standing among his brother practitioners? There is need in the future for thorough and high class society work, and, fortunately, under the new order of civic laws, the way is made easier and less expensive than in the past. Our first duty is to attend and support our State society meeting in the spring at an expense of two dollars a year. The next outlay will be at the district meetings in the fall. It should be a pleasure to attend both these meetings, and there are none so poor but they can do so if they wish.

There is another duty that has been overlooked and neglected by too many in the profession, and it would seem as if their conscience would prick them when they read the reports in the journals of the meetings of the American Dental Association. While many may feel that they cannot attend the summer meetings of the Association regularly, they ought to have enough interest in it to help it along in its good work. It would startle even those most interested were they to recall even a part of the valuable work that has been done by the members of the Association, and which has been freely given out broadcast to the profession. There are many in this district that can easily afford to become permanent members of the Association, and they should willingly pay the yearly dues of five dollars to help the Association along in the work it has been doing since its organization. The amount of dues to the three associations is less than ten dollars a year, a very small part of the yearly income to pay out for the new ideas and valuable information we may get in return from attending these meetings, especially the American Dental Association, where we may have an opportunity to meet and make new acquaintances and keep in touch with the prominent men in the profession from nearly all parts of the country. There has been no sectional feeling in the American Dental Association. The best efforts of its members have been exerted in a line of work that will benefit the profession of the world. The Association has been restricted in the past from lack of members and consequent lack of funds. If a goodly number of the men who have in years past attended the meetings as delegates had continued on as permanent members, the financial condition would have been such that much more of good might have been accomplished. There are said to be over twenty thousand dentists in the United States who are legally qualified to practise. They each and every one profess to be dentists, and yet it is a lamentable fact that of the entire number the total membership of the American Dental Association for the past two years has been less than two hundred and fifty members; and of this number cultured New England, under the most favorable circumstances and conditions, furnished only seventeen members. If only one in twenty of the dentists of the country could be induced to become permanent paying members of the Association, it would soon be in condition to co-operate in a satisfactory manner with the curator of the Army Medical Museum in Washington, D. C., who stands ready and anxious to help along the movement recently inaugurated by the American Dental Association, and to place in the rooms already set apart for the dental department everything of value that can be secured in the future in the way of books, specimens, and materials that will be of interest to the dental profession. It is time to attempt to arouse a more general interest in professional work and to start a dental revival, as there is a pressing need for missionary work to be done all over the country, and especially in certain districts, that can best be influenced by the workers in the State societies. The promoters of this Association, while loyal to their State organizations, are equally anxious and desirous that the State societies should join with them in their efforts to make the Northeastern Dental Association a successful post-graduate school. With the hearty co-operation of the dentists of this district, we may reasonably hope to benefit and to accomplish for the profession of New England all that it has been the aim of the American Dental Association to do in the past for the profession of the entire country.

